# Generation and Characterization of SORT1-Targeted Antibody–Drug Conjugate for the Treatment of SORT1-Positive Breast Tumor

**DOI:** 10.3390/ijms242417631

**Published:** 2023-12-18

**Authors:** Weiliang Zhuang, Wei Zhang, Liping Xie, Lei Wang, Yuan Li, Ziyu Wang, Ao Zhang, Haitao Qiu, Jun Feng, Baohong Zhang, Youjia Hu

**Affiliations:** 1 Engineering Research Center of Cell & Therapeutic Antibody, Ministry of Education, School of Pharmacy, Shanghai Jiao Tong University, 800 Dongchuan Road, Shanghai 200240, China; zhuangweiliang@sjtu.edu.cn (W.Z.); lwangph@sjtu.edu.cn (L.W.); 2 China State Institute of Pharmaceutical Industry, 285 Gebaini Road, Shanghai 201203, China; zhangwei31@sinopharm.com (W.Z.); xieliping@sinopharm.com (L.X.); liyuan21705006@163.com (Y.L.); crushziyu@163.com (Z.W.); zhangao9907@163.com (A.Z.); qhtygy2021@163.com (H.Q.); fengjdmr@163.com (J.F.)

**Keywords:** SORT1, ADC, MMAE, DXd, internalization, breast cancer

## Abstract

Antibody–drug conjugates (ADCs) have greatly improved the outcomes of advanced breast tumors. However, the treatment of breast tumors with existing ADCs is still hindered by many issues, such as tumor antigen heterogeneity and drug resistance. Therefore, ADCs against new targets would provide options for the treatment of these challenges. Sortilin-1 (SORT1) may be a promising target for ADC as it is upregulated in breast cancer. To evaluate the possibility of SORT1 as an ADC target, a humanized antibody_8D302 with high affinity against SORT1 was generated. Additionally, 8D302 was conjugated with MMAE and DXd to generate two ADCs_8D302-MMAE and 8D302-DXd, respectively. Both 8D302-MMAE and 8D302-DXd showed effective cytotoxicity against SORT1 positive breast tumor cell lines and induced bystander killing. Consequently, 8D302-MMAE showed relatively better anti-tumor activity than 8D302-DXd both in vitro and in vivo, but 8D302-DXd had superior safety profile and pharmacokinetics profile over 8D302-MMAE. Furthermore, SORT1 induced faster internalization and lysosomal trafficking of antibodies and had a higher turnover compared with HER2. Also, 8D302-DXd exhibited superior cell cytotoxicity and tumor suppression over trastuzumab-DXd, a HER2-targeted ADC. We hypothesize that the high turnover of SORT1 enables SORT1-targeted ADC to be a powerful agent for the treatment of SORT1-positive breast tumor.

## 1. Introduction

Developing antibody–drug conjugates (ADCs) through coupling cytotoxic agents to antibodies has often proven to be a better therapeutic option for treating cancer compared to using antibodies alone, and more than 10 ADC agents have already been approved for clinical use [1]. In this regard, ADCs used in breast cancer treatment include Kadcyla and Enhertu, targeting human epidermal growth factor receptor 2 (HER2) [2], and Trodelvy, targeting trophoblast cell surface antigen-2 (TROP2) [3]. However, because of the heterogeneity of tumor antigens, existing ADCs do not cover all types of breast cancer patients. For example, only about 20–30% breast cancers overexpress HER2 [4], and Trop-2 is mainly overexpressed in triple negative breast cancer (TNBC) when compared to other types of breast cancers. In addition, ADCs are not effective for all patients even with target-high expression on tumor [5]. The overall response rate (ORR) of Kadcyla (T-DM1) for the treatment of HER2-positive metastatic breast cancer that had been previously treated with trastuzumab and a taxane was only 34.2%, whereas Enhertu (DS-8201a) was relatively effective with the ORR of 79.7% in the same clinical trial [2,6]. The overall response rate of Trodelvy for the treatment of TROP2 high expression cancer and low expression cancer were only 44% and 22% [7], respectively. Furthermore, breast cancer patients often develop resistance against existing ADCs due to the downregulation of the target or the upregulation of drug efflux transporters [8]. Therefore, new target ADCs for the treatment of breast cancer are worth studying to meet any unmet medical needs. Currently, many ADCs targeting different antigens have been utilized within clinical trials focusing on breast cancer treatment; these targets include but are not limited to HER3, Nectin-4, ROR1, B7H4, and LIV-1 [9]. This study aimed to explore the potential of SORT1-targeted ADCs in the treatment of breast cancer.

SORT1, also known as neurotensin receptor-3 (NT3), is a member of the vacuolar protein sorting 10 (VPS10) family which is involved in various biological processes [10]. SORT1 is a multiligand receptor that plays different roles in transporting and sorting various ligands, including neurotensin, progranulin, and apolipoprotein [11,12,13]. SORT1 is associated with many pathological diseases, including Alzheimer’s disease [14], cardiovascular disease [15], and type 2 diabetes [16]. On the other hand, SORT1 was upregulated in many types of human cancer, such as breast, ovarian, pancreatic, melanoma, and pituitary adenoma [17,18]. This suggests that SORT1 is a potentially therapeutic target for tumor therapy. Theratechnologies developed a SORT1-targeted peptide–drug conjugate (PDC)_TH1902 for the treatment of SORT1-positive triple-negative breast cancer [19]. TH1902 was generated by a conjugated TH1901 peptide with two molecules of docetaxel and demonstrated significant tumor-suppressive activity in triple-negative breast cancer xenograft models. This means that SORT1-targeted ADCs may also have efficacy against triple-negative breast cancer.

In the present study, we generated a novel humanized anti-SORT1 antibody-8D302. To investigate the potency of SORT1 as an ADC target, we used 8D302 to develop two ADC drugs with different payloads and linkers. As expected, 8D302-DXd and 8D302-MMAE showed superior antitumor efficacy over isotype-ADCs and 8D302, and 8D302-DXd revealed a relatively better safety profile when compared to 8D302-MMAE. We also found that 8D302-DXd induced superior cell cytotoxicity against T47D and MDA-MB-231 and tumor suppression in MDA-MB-231 xenograft model over trastuzumab-DXd. The results of confocal microscopy and flow cytometry demonstrated that SORT1 facilitated more SORT1-antibodies to be internalized into lysosome, which would provide the SORT1-targeted ADC with better efficacy.

## 2. Results

### 2.1. Generation and Identification of Humanized Anti-SORT1 Antibody

**Figure 1:**
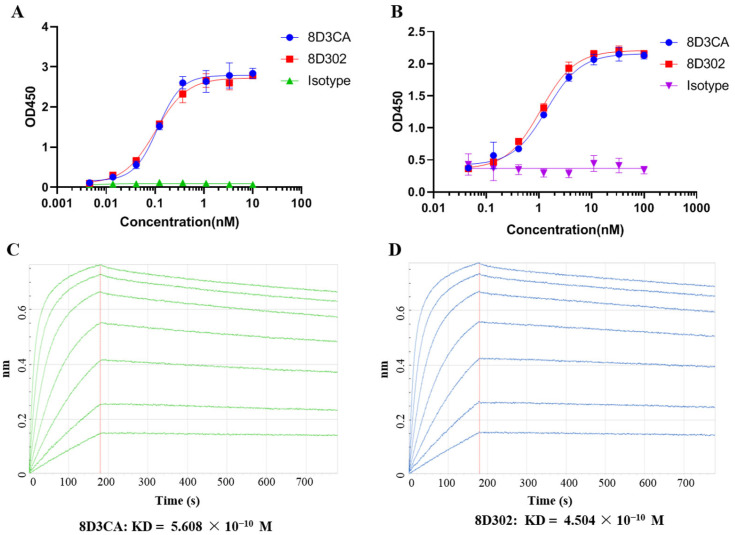
Characterization of anti-SORT1 humanized antibody_8D302. (**A**) The binding activity of 8D302 and 8D3CA against SORT1 ECD protein was detected by ELISA. (**B**) Cell-binding activity of 8D302 and 8D3CA was evaluated by cell-based ELISA. EC_50_ values were calculated by GraphPad Prism software 8.0. Data are presented as mean ± SD from 3 independent experiments. (**C**,**D**) Affinity of 8D3CA (**C**) and 8D302 (**D**) was measured using BLI technology. The red line indicates the dividing point between binding and dissociation.

The murine anti-SORT1 antibodies were obtained by hybridoma technology. After a series of in vitro screenings, 8D3 was selected as a candidate of murine anti-SORT1 antibodies. The variable regions of 8D3 were linked with corresponding constant regions of human IgG1 to construct the chimeric antibody_8D3CA. To reduce immunogenicity, 8D3 was humanized using CDR-grafting technology, followed by further optimization with back mutation [20]. As a result, the protein-binding activity and cell-binding capacity of the optimal humanized SORT1 antibody_8D302 were comparable to 8D3CA (Figure 1). The affinity KD value of 8D3CA and 8D302 were 5.608 × 10^−10^ M and 4.504 × 10^−10^ M, respectively. These results demonstrated that humanization did not impact the binding affinity of 8D302 to SORT1.

**Figure 2:**
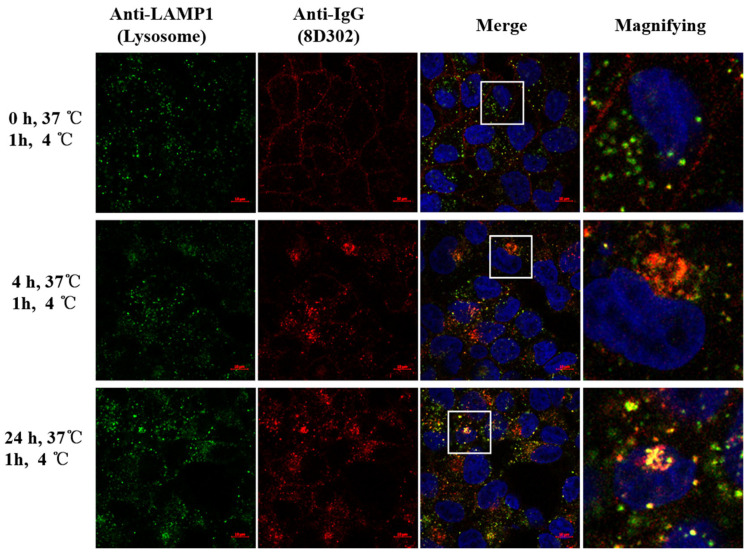
Internalization of 8D302 was detected using confocal microscopy. T47D cells were incubated with 8D302 for 0, 4, or 24 h at 37 °C, followed by incubation at 4 °C for 1 h. After fixation and permeabilization, lysosomes were localized with anti-lamp1 antibodies followed by being stained with Alexa Fluor 488-conjugated secondary antibodies (green), SORT1 antibodies (8D302) were stained with Alexa Fluor 647-conjugated secondary antibodies (red), and the nucleus were labeled with DAPI (blue). The white frame areas of pictures were magnified on the right.

Some previous studies demonstrated that SORT1 induces endocytosis and transports its ligand from extracellular region into intracellular lysosome [11,12,21]. Therefore, we investigated if SORT1 could induce endocytosis of 8D302 into SORT1-positive cancer cells. As shown in Figure 2, 8D302 was only detectable on the surface of T47D cells initially. After incubation at 37 °C for 4 or 24 h, lots of 8D302 were internalized in the intercellular and colocalized with lysosomes.

### 2.2. Conjugation of 8D302

**Figure 3:**
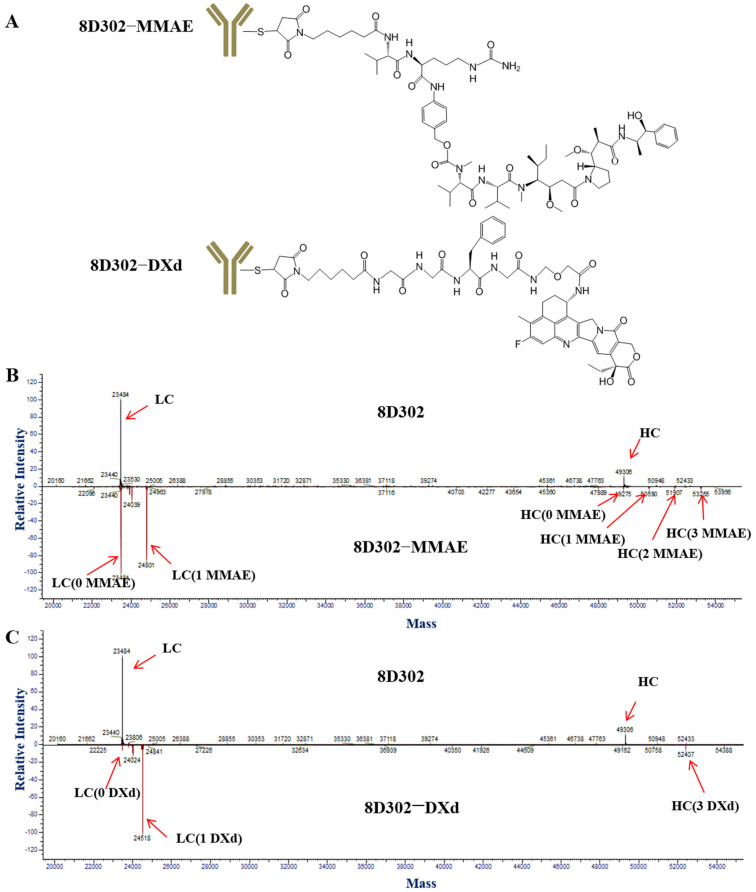
Characterization and structures of 8D302-MMAE and 8D302-DXd. (**A**) The molecular structures of 8D302-MMAE and 8D302-DXd. Here, 8D302 were controllably reduced using TCEP, followed by incubation with the drug. Conjugates were formed by a covalent reaction between the maleimidocaproyl on the drugs and free sulfhydryl groups on the antibodies. (**B**,**C**) DAR values of 8D302-MMAE (**B**) and 8D302-DXd (**C**) were detected by the LC–MS analysis.

To choose the suitable toxic payload for the conjugation of 8D302, we selected MMAE and DXd as candidates due to the fact that they had different cytotoxic mechanisms [22]. MMAE causes cell death in dividing cells by inhibiting tubulin polymerization. DXd hampers DNA replication and triggers apoptotic cell death by inhibiting the relegation of broken DNAs. Also, 8D302-MMAE was acquired by covalent conjugation of MMAE on antibody cystine groups (Figure 3A). The linker of 8D302-MMAE was valine-citrulline (VC) dipeptide which was selective cleavage by cathepsin B. The DAR value of 8D302-MMAE was 3.98 (Figure 3B), similar to the MMAE-coupled ADCs that are on the market [23]. The conjugation method of 8D302-DXd was the same as that of DS-8201a [24]. DXd was conjugated on the cysteine residue of 8D302 via a glycyl-glycyl-phenylalanyl-glycyl (GGFG) peptide linker which was selectively cleaved by lysosomal enzymes such as cathepsin B and cathepsin L. The result (Figure 3C) showed the DAR value of 8D302-DXd was 7.57. As the negative control, isotype-MMAE and isotype-DXd were prepared by conjugating isotype antibody with MMAE and DXd, respectively. The DAR values of isotype-MMAE and isotype-DXd were 4.01 and 7.72, respectively.

As shown in Figure S1, the binding activity of 8D302-ADCs was approximately at the same level as 8D302. This demonstrated that conjugation with payloads did not significantly affect the affinity of 8D302 against SORT1.

### 2.3. Evaluation of 8D302-ADCs Anti-Tumor Effects In Vitro

**Figure 4:**
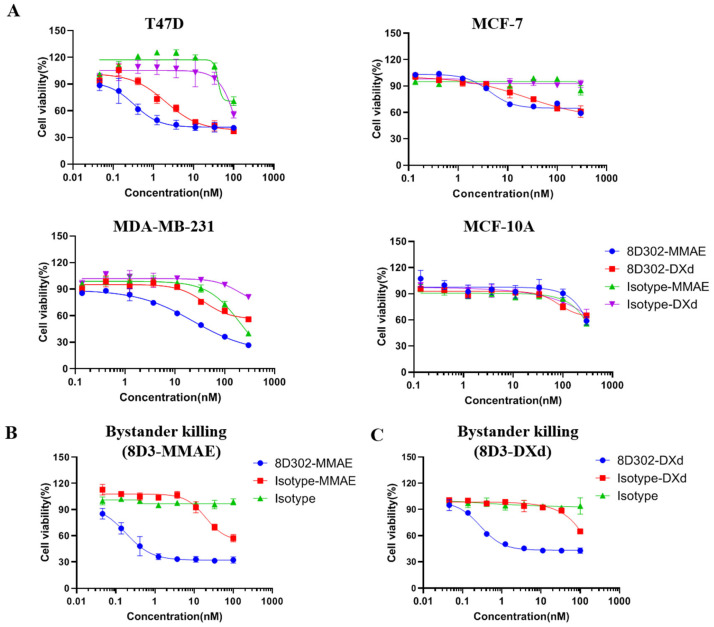
The cytotoxicity activity and bystander killing activity of ADCs. (**A**) Comparison of the cytotoxicity activity between 8D302-MMAE and 8D302-DXd against T47D, MCF-7, MDA-MB-231, and MCF-10A. Serially diluted ADCs were added to the cells. After incubation for 72 h, cell viability was detected using CCK8. (**B**,**C**) Bystander killing activity of 8D302-MMAE (**B**) and 8D302-DXd (**C**). Serially diluted ADCs were added to the CHO-K1/SORT1 for 48 h. The cell supernatants were then added to Expi293F for incubation another 48 h, followed by detection of cell viability using CCK8. Data are presented as mean ± SD from 3 independent experiments. The IC_50_ value was calculated using GraphPad Prism 8.0 software.

SORT1 expression on the surface of the human breast cell lines T47D, MCF-7, MDA-MB-231, and MCF10A was evaluated by flow cytometry. From Figure S2, we found that SORT1 was expressed on the surfaces of breast tumor cells T47D, MCF-7, and MDA-MB-231, but was not present on MCF-10A, a non-tumor breast cell line. Cell cytotoxicity was performed by incubating cells with different ADCs for 72 h. Cell viability was analyzed using CCK8 assay. As shown in Figure 4, T47D was sensitive to 8D302-MMAE and 8D302-DXd with IC_50_ values of 0.33 nM and 2.05 nM, respectively. Meanwhile, 8D302-MMAE and 8D302-DXd induced relative weaker cell cytotoxicity on MCF-7 with IC_50_ values of 4.3 nM and 24.07 nM, respectively. The 8D302-MMAE induced growth inhibition on MDA-MB-231 with IC_50_ values of 23.28 nM, while 8D302-DXd had relatively weaker cytotoxic effect. Furthermore, all three tumor cell lines were more sensitive to 8D302-MMAE than 8D302-DXd. Both 8D302-MMAE and 8D302-DXd did not have obvious cytotoxicity against SORT1-negative cell MCF-10A. These results suggested that SORT1-targeted ADC had cytotoxicity on SORT1-positive tumor cells in vitro and MMAE may be the more effective payload for SORT1-targeted ADC to kill tumor cells.

In order to investigate whether SORT1-targeted ADCs killed neighboring cells, a bystander killing assay was carried out. As shown in Figure 4B,C, both 8D302-MMAE and 8D302-DXd induced bystander killing against Expi293. Our preliminary data showed that Expi293 was insensitive to SORT1-targeted ADCs. These results were in line with the outcomes of previously published ADCs which had the same payloads and linkers [25,26].

### 2.4. Anti-Tumor Efficacy of 8D302-ADCs In Vivo

**Figure 5:**
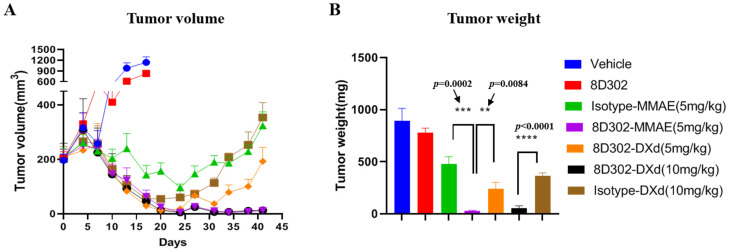
Anti-tumor efficacy of ADCs in MDA-MB-231 xenograft model. The tumor-bearing M-NSG mice were injected via intravenous with samples on day 0 and day 7. Tumor volume (**A**) was measured twice a week. Tumor weight (**B**) was measured at the end of study. The data are presented as mean ± SEM from five mice. **, *p* < 0.01, ***, *p* < 0.001, ****, *p* < 0.0001.

We analyzed the anti-tumor effects of 8D302-ADCs using the MDA-MB-231 breast tumor model in M-NSG mice. After two doses of 5 mg/kg or 10 mg/kg, all ADCs induced significant tumor suppression compared to the vehicle control, while 8D302 alone had weak efficacy on tumor inhibition (Figure 5). Furthermore, 8D302-MMAE (5 mg/kg) and 8D302-DXd (10 mg/kg) showed comparable tumor inhibitory activity, and their efficacy was superior to their corresponding isotype-ADCs at the same dose. The non-binding isotype-ADCs also showed some antitumor activity. This may be due to the bystander killing of drugs which was extracellularly cleaved from the ADC in the tumor environment. Consistent with the results of in vitro studies, the tumor inhibition induced by 8D302-MMAE at two doses of 5 mg/kg was significantly better than that of 8D302-DXd (5 mg/kg). Importantly, no significant change in mice body weight was observed in any of the treatment groups, indicating that 8D302-ADCs might be well tolerated at the testing dose (Figure S3).

### 2.5. Safety and Pharmacokinetics of 8D302-ADCs in Mice

**Figure 6:**
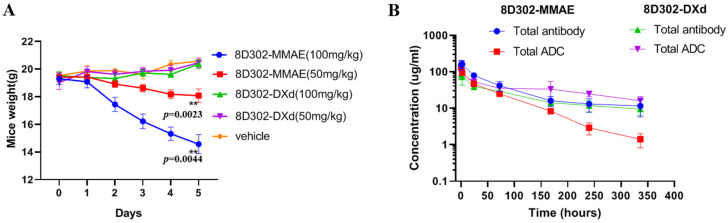
Acute toxicity and pharmacokinetics of ADCs. (**A**) The acute toxicity of 8D302-MMAE and 8D302-DXd in mice. BALB/c mice in groups (n = 5 per group) were injected via intravenous with one dose (50 mg/kg or 100 mg/kg) of ADCs. Mice weight and mortality were observed every day. The data are presented as mean ± SEM from five mice. **, *p* < 0.01. (**B**) The pharmacokinetics of 8D302-MMAE and 8D302-DXd in mice. BALB/c mice in two groups (n = 3 per group) were injected in vitro with one dose (5 mg/kg) of 8D302-MMAE and 8D302-DXd, respectively. The serum samples were collected at 0.1, 2, 24, 72, 168, 240, and 336 h after injection. The concentration of total antibodies and total ADCs were detected using ELISA. The t1/2 values were calculated using PKslover 2.0 software. The data are presented as mean ± SEM from three mice.

As mentioned above, both 8D302-MMAE and 8D302-DXd did not affect the weight of the mice at the testing dose. To further investigate the safety of two 8D302-ADCs in vivo, an acute toxicity study with a single dose (50 and 100 mg/kg) of 8D302-MMAE and 8D302-DXd was evaluated in BALB/c mice. As shown in Figure 6A, both doses of 8D302-MMAE caused body weight loss in mice, and all mice with a 100 mg/kg dose of 8D302-MMAE died within 6 days. However, no weight loss and death events were observed in 8D302-DXd at the same dose. These results indicated that the 8D302-DXd had a better safety profile than the 8D302-MMAE, and the maximal toxic dose (MTD) of 8D302-DXd in mice was more than twice that of 8D302-MMAE. Although 8D302-DXd was not as effective as 8D302-MMAE at the same dose, its safety profile would contribute to a wider therapeutic window.

The pharmacokinetics profile was evaluated in BALB/c mice with a 5 mg/kg dose of 8D302-MMAE or 8D302-DXd. As shown in Figure 6B, the pharmacokinetic profiles of 8D302-MMAE, 8D302-DXd antibody and 8D302-DXd total ADC were almost consistent. However, the total ADC of 8D302-MMAE concentration in the serum decreased faster with the mean half-life time (t1/2) of 62.3 h, while the mean t1/2 of the total antibodies of 8D302-MMAE was 146.9 h. The total of ADCs and of antibodies 8D302-DXd have relatively long half-lives with the mean t1/2 of 168 h and 222.9 h, respectively. This result demonstrated that 8D302-DXd was more stable than 8D302-MMAE in mice and MMAE was detached sooner from the ADC during metabolism, which may be one of the reasons why 8D302-MMAE was more toxic in vivo.

### 2.6. Comparison of Internalization Profile between SORT1 and HER2

**Figure 7:**
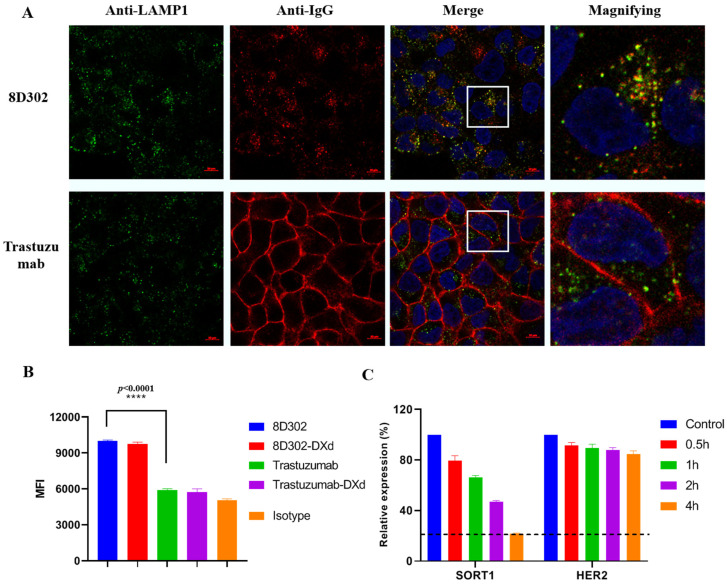
Comparison of internalization profile between SORT1 and HER2. (**A**) Internalization of SORT1 antibody and HER2 antibody were detected using confocal microscopy. T47D cells were incubated with 8D302 (10 nM) or trastuzumab (10 nM) for 4 h at 37 °C, followed by fluorescent labeling. lysosomes were localized with anti-lamp1 antibodies followed by being stained with Alexa Fluor 488-conjugated secondary antibodies (green), 8D302 or trastuzumab were stained with Alexa Fluor 647-conjugated secondary antibodies (red); and the nucleus were labeled with DAPI (blue). The white frame areas of pictures were magnified on the right. (**B**) The detection of antibodies and ADCs’ internalization using flow cytometry. T47D cells were treated with pHrodo^TM^ green-labeled antibodies (10 nM) for 24 h. *p* value was calculated between 8D302 and trastuzumab using *t*-test analysis. ****, *p* < 0.0001. (**C**) Flow cytometry analysis of antigen levels on cell surface. T47D cells were treated with monensin (50 uM) for 0.5, 1, 2, or 4 h at 37 °C. Cells were incubated with SORT1 antibody or HER2 antibody at 4 °C for 1 h, followed by staining with Alexa Fluor 647-conjugated goat anti-Human IgG for 0.5 h. MFI of Alexa Fluor 647 was measured on a flow cytometer. Surface protein levels were expressed as a relative percentage of untreated cells. Data are presented as mean ± SD from 3 independent experiments.

Previous reports indicated that ADC induces superior cytotoxicity if its target undergoes rapid internalization [26,27,28]. To evaluate the internalization properties of SORT1, we chose HER2 as a reference, which does not rapidly internalize [29]. The internalization assay was performed by confocal microscopy using breast cancer cell T47D which expressed more HER2 on the cell surface than SORT1 (Figure S2). After incubation for 4 h, SORT1 antibodies (8D302) were evidently internalized and partly colocalized to lysosomes, whereas HER2 antibodies (trastuzumab) remained on the cell surface (Figure 7A). We also confirmed this internalization result using pHrodo^TM^ iFL green-labeled antibodies whose fluorescence could only be detected under low pH condition, such as lysosomes or endosomes. After incubation for 24 h, the mean fluorescence intensity (MFI) of pHrodo^TM^ iFL green was detected by a flow cytometry. As shown in Figure 7B, the MFI of 8D302-treated cell was significantly higher than that of trastuzumab, indicating that more 8D302 was transported to lysosomes compared with trastuzumab. To compare the internalization of ADCs, we constructed an HER2-targeted ADC_trasuzumab-DXd which had a DAR value of 7.42. Additionally, 8D302-DXd and trasuzumab-DXd showed a similar internalization profile as their non-conjugated antibodies, which demonstrated that conjugation had no effect on antibody internalization. Taken together, these results indicated that SORT1 could induce more internalization and lysosomal trafficking of antibodies under lower surface expression.

SORT1 is located in both cell surfaces and cytoplasm. It shuttles among the cell surface, lysosome, and the Golgi apparatus to transport various proteins. On the contrary, HER2 is majorly localized to the cell surface. The expression profile and function of SORT1 suggest that it is a high turnover protein, which could explain why SORT1 mediates more antibody internalization. To prove this hypothesis, monensin was added to block cell surface trafficking of proteins from intracellular, then the remaining protein on the surface was detected using flow cytometry. The level of SORT1 on the T47D surface rapidly decreased with the treatment of monensin. After incubation for 4 h, the level of surface SORT1 was downregulated by about 80%, while only about a 15% reduction was found on surface HER2. This result indicated that SORT1 had a high turnover with continuous internalization and supplementation from intracellular.

### 2.7. Comparison of Anti-Tumor Effects between SORT1-Targeted ADC and HER2-Targeted ADC

**Figure 8:**
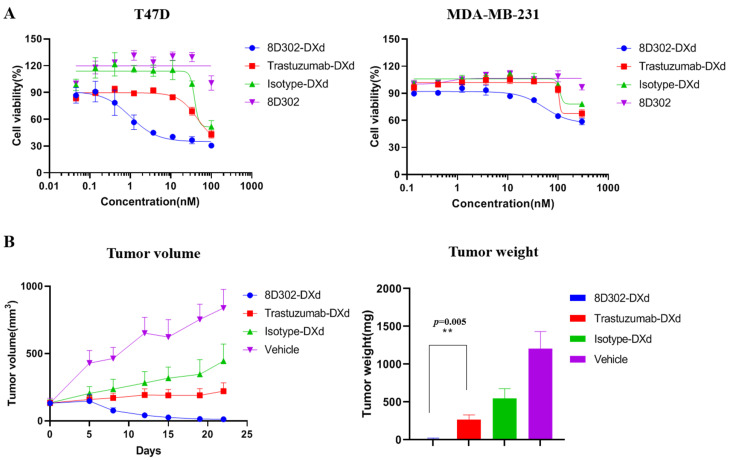
The anti-tumor effects of 8D302-DXd and trastuzumab-DXd. (**A**) Comparison of cytotoxicity activity between 8D302-DXd and trastuzumab-DXd on T47D and MDA-MB-231. The IC_50_ value was calculated using GraphPad Prism software. Data are presented as mean ± SD. (**B**) Comparison of tumor suppressor activity between 8D302-DXd and trastuzumab-DXd in MDA-MB-231 xenograft model. The tumor-bearing BALB/c nude mice were injected via intravenous with a single dose of vehicle, 8D302-DXd (10 mg/kg), trastuzumab-DXd (10 mg/kg), or isotype-DXd (10 mg/kg). Data are presented as mean ± SEM. **, *p* < 0.01.

To investigate if the internalization profile of SORT1 would provide SORT1-targeted ADCs with a more powerful anti-tumor effect, we compared the cell cytotoxicity between SORT1-targeted ADCs and HER2-targeted ADCs using 8D302-DXd and trastuzumab-DXd, a biosimilar of DS-8201a. DS-8201a was proved to notably inhibit the growth of HER2-positive tumors in preclinical and clinical environments [30,31]. In the T47D and MDA-MB-231, the surface levels of HER2 were higher than that of SORT1 (Figure S2). However, 8D302-DXd induced cell cytotoxicity against T47D and MDA-MB-231 with IC_50_ of 1 nM and 53.43 nM (Figure 8), respectively, while trastuzumab-DXd had a weak effect. We also evaluate the in vivo anti-tumor effects of 8D302-DXd and trastuzumab-DXd using an MDA-MB-231 xenograft model. As expected, 8D302-DXd showed significantly superior efficacy in inhibiting tumor growth when compared to trastuzumab-DXd (Figure 8). These results indicated that the internalization induced by SORT1 would enable more ADCs into cells and provide ADCs with more powerful tumor suppressive activity.

## 3. Discussion

In the last decade, breast cancer therapy has been greatly improved with the approval of multiple new drugs, such as small molecule novel target inhibitors, immunotherapy antibodies, and ADCs [32]. ADCs have shown impressive outcomes for treatment of breast cancer. Kadcyla and Enhertu were approved by the FDA for patients with advanced or metastatic HER2-positive breast cancer in 2013 and 2019 [2], respectively. Trodelvy was approved by the FDA in 2021 for treatment of metastatic TNBC patients who had received ≥2 prior systemic therapies [9]. However, these ADCs do not meet all clinical needs due to tumor antigen heterogeneity and drug resistance. Developing new target ADCs is an effective way to address these challenges. Many ADCs targeting new antigens are currently under investigation. HER3, Nectin-4, ROR1, LIV-1, and B7–H4 ADCs have shown encouraging results in clinic [9]. In addition, payloads and linkers can also address tumor antigen heterogeneity and drug resistance [33]. The same antibody conjugated with different payloads and linkers usually exhibited different therapeutic efficacy and safety characteristics. Compared with T-DM1, DS-8201a has cleavable linkers and more efficient cytotoxic agents [25]. DS-8201a was not only effective against T-DM1-resistant breast cancer [34], but also exhibited stronger tumor suppressive activity in HER2-low tumors, on which T-DM1 had no effect [24]. In this study, we developed two SORT1-targeted ADCs with different payloads for the treatment of breast tumor. On the one hand, we are trying to investigate the potential of SORT1 as an ADC drug target; on the other hand, we are trying to investigate the suitable payload for SORT1-targeted ADC.

Previous studies had shown that SORT1 was overexpressed in breast cancer cells and underexpressed in normal cells [17]. Our results demonstrated that the surface of breast cancer cells was localized with a large amount of SORT1, while it was not present on the surface of non-tumor breast cell_MCF-10A. The expression profile supported SORT1 as a potential target for new ADC development. In addition, a SORT1-targeted PDC-TH1902 is now evaluated in clinical environments to treat TNBC. The PDC demonstrated significant efficacy and good safety in preclinical studies [19]. However, TH1902 required multiple high-dosage injections to completely inhibit the growth of tumor growth in MDA-MB-231 xenograft mice. On the contrary, 8D302-DXd showed complete inhibition at one dose of 10 mg/kg. This may be due to relatively short half-life of PDC in vivo. TH1902 has only 1.44 h of half-life in mice, while the half-life of ADC in mice is usually more than 48 h [35]. In this study, the half-life of 8D302-DXd and 8D302-MMAE were 62.3 and 222.9 h, respectively. Furthermore, 8D302-DXd exhibited brilliant a safety profile with no significant effect on mice body weight at a dose of 100 mg/kg. This suggests that ADC may be a better form for SORT1-targeted drugs compared with PDC.

Currently, the most commonly used toxins for killing tumor cells are microtubule inhibitors and DNA inhibitors [22]. Microtubule inhibitors which were commonly used in ADCs included MMAE, MMAF, DM1, and DM4. DNA inhibitors for ADCs included DXd, SN38, and PBD. From the two types of toxin, we selected MMAE and DXd as payloads, respectively. MMAE induced superior cytotoxicity on several cancer cell lines with the IC_50_ values range from 0.06 to 1.48 nM [36], while DXd showed relatively moderate cytotoxicity on tumor cells with IC_50_ values of 1.43–4.07 nM [24]. As expected, all breast tumor cells in the study were more sensitive to 8D302-MMAE than 8D302-DXd. In vivo data also confirmed the result that tumor growth inhibition induced by 8D302-MMAE was better than 8D302-DXd. However, previous reports demonstrated that DXd-conjugated ADCs exhibited better safety characteristics than MMAE ADCs in both human and animal models. For example, the MTD of RC48-ADC (a HER2-targeted ADC) was 2.5 mg/kg in a phase I study in patients [37], while the MTD in patients of DS-8201a was more than 8 mg/kg [34]. In addition, the toxicological results of TROP2-targeted ADCs in monkeys showed that DXd-conjugated ADCs have the best safety profile. The MTD of SY02-DXd was more than six times that of SY02-MMAE [38]. Similarly, we found that 8D302-DXd exhibited a superior safety profile in mice over 8D302-MMAE. Since 8D302 does not bind to murine SORT1, targeted toxicity of 8D302-ADCs needs to be further studied in the future. Furthermore, 8D302-DXd showed longer high-life in mice when compared with 8D302-MMAE, suggesting that the dose interval of 8D302-DXd could be longer. The shorter high-life of 8D302-MMAE may be due to the fact that the VC linker was not as stable as the GGFG linker in mouse blood. Both the VC linker and the GGFG linker are cleaved by lysosomal enzymes in tumor and stable in human serum [24,39], but the VC linker has been reported to be sensitive to extracellular carboxylesterases in mouse serum [39]. Therefore, the pharmacokinetics profile of 8D302-MMAE and 8D302-DXd need to be further studied in other animal models. 

The efficacy of ADC not only depends on linkers and payloads, but the expression level and internalization profile of tumor targets make great contributions as well [40]. We found that the surface expression level of SORT1 on T47D were lower than that of HER2. However, more SORT1 antibodies were internalized and targeted lysosomes when compared with HER2 antibodies. The faster downregulation of surface SORT1 when protein-trafficking was blocked demonstrates that SORT1 was a high-turnover protein, which could explain why more SORT1 antibodies were internalized. Similar results were previously found at other targets which also induced rapid internalization and had a high turnover, such as PRLR, HER3, and TF [26,27,28]. PRLR-targeted ADCs and HER3-targeted ADCs are now in clinical trials for patients with advanced solid tumors [41,42]. TIVDAK, a TF-targeted ADC, had been approved by the FDA for recurrent or metastatic cervical cancer [43]. These suggest that SORT1-targeted ADCs may be a promising agent for SORT1-positve cancers in clinic.

To further study whether rapid internalization benefited SORT1-targeted ADCs, we compared the tumor suppressive activity of 8D302-DXd and trastuzumab-DXd both in vitro and in vivo. As expected, the antitumor activity of 8D302-DXd induced better cytotoxicity against T47D and MDA-MB-231 compared to trastuzumab-DXd. In MDA-MB-231 xenograft model, the tumor suppressive activity of 8D302-DXd was significantly better than trastuzumab-DXd. MDA-MB-231 was considered as a HER2-low expression breast cancer cell, so whether 8D302-DXd is still more effective in HER2-high expression breast cancer needs further evaluation. DS-8201a showed efficacy in patients with HER2-low or no expression, but its response rate was lower than that of patients with HER2-high expression [31]. Therefore, SORT1-targeted ADCs may be more effective than HER2-targeted ADCs for the treatment of SORT1-positive and HER2-negative or low expression cancers.

## 4. Materials and Methods

### 4.1. Cell Lines

Myeloma cell line SP2/0, Chinese hamster ovary cell line CHO-K1, and human breast cell lines (T47D, MDA-MB-M468, MDA-MB-231, MCF-7, and MCF-10A) were all obtained from ATCC. A SORT1 overexpression cell line, designated as CHO-K1/SORT1, was obtained by transfecting CHO-K1 with SORT1 expression plasmid and stable selection using hygromycin. SP2/0, MDA-MB-M468, and MDA-MB-231 were cultured in DMEM (Hyclone, Logan, UT, USA) containing 10% fetal bovine serum (FBS) (Gibco, Waltham, MA, USA). CHO-K1 and CHO-K1/SORT1 were cultured in DMEM/F12 (Hyclone, Logan, UT, USA) containing 10%FBS. T47D were cultured in RPMI-1640 (Hyclone, Logan, UT, USA) containing 10% FBS. MCF-7 was cultured in MEM (Hyclone, Logan, UT, USA) containing 10% FBS. MCF-10A was cultured in MCF-10A complete medium (Procell, Foshan City, China). Expi293F was purchased from Gibco and cultured in Expi293 medium (Gibco, Waltham, MA, USA). 

### 4.2. Antibodies and Conjugates 

To generate the SORT1 antibody, BALB/c mice were immunized with SORT1 protein on day 0, day 14 and day 35, followed by booster immunization using CHO-K1/SORT1 cell on day 56 and last injection with SORT1 protein on day 77. The spleen cells of immunized mice were later fused with the myeloma cell line SP2/0. The positive hybridoma cells, secreting SORT1-specific antibodies, were screened through two rounds of enzyme-linked immune sorbent assays (ELISA). This was followed by further sub-cloning of positive clones. The variable region sequence of SORT1 murine antibody was obtained from positive hybridoma cells using RT-PCR, and the humanized antibody was constructed by complementary determining region (CDR) grafting and back-mutation. In addition, trastuzumab, an HER2 antibody, was used as a reference antibody in this study. The heavy and light chain sequences of trastuzumab are acquired from international ImMunoGeneTics information system (IMGT). A non-binding isotype antibody was used as the negative control in the study. The expression plasmid of antibodies was constructed based on the mammalian expression vector pCDNA3.1. The antibodies were further expressed by transfection with corresponding plasmids in Expi293F cells and purified by protein A affinity chromatography. 

MMAE-conjugated ADCs were prepared as previously described [44]. The antibody partially reduced via incubating with tris (2-carboxyethyl)-phosphine (TCEP) (MCE, Buford, GA, USA) at 37 °C for 2 h. The molar concentration ratio of TCEP to antibody was 4:1. An eight-fold molar excess of MMAE (MCE, Buford, GA, USA), which contained a VC linker and maleimidocaproyl moiety, was added to the reduced antibodies for 1 h on ice. The ADCs were subsequently purified by protein A affinity chromatography.

To prepare the DXd (MCE, Buford, GA, USA) conjugated ADCs, the antibody was incubated with TCEP at 37 °C for 2 h. The molar concentration of TCEP was eight times that of antibodies in order to completely reduce the interchain disulfide bonds of antibody. DXd containing a GGFG linker and maleimidocaproyl moiety was added to the reduced antibodies for 1 h on ice. The molar concentration ratio of DXd to antibodies was 15:1. The ADCs were further purified by protein A affinity chromatography to remove unconjugated DXd.

### 4.3. Binding Assay

For protein binding, 1 µg/mL SORT1 extracellular domain (ECD) protein were directly coated onto 96-well immunoplates (Thermo Fisher, Waltham, MA, USA) and incubated overnight at 4 °C. After washing, the plate was blocked with 1% BSA at 37 °C for 1 h. Serial diluted antibodies, ranging from 10 nM to 4.6 pM, were then added in triplicates. After incubation at 37 °C for 1 h, the plate was washed and incubated with horseradish peroxidase (HRP) conjugated goat anti-human IgG (Sigma, Taufkirchen, Germany) at 37 °C for 0.5 h. After washing, 3,3′,5,5′-tetramethylbenzidine (TMB) was added for 5 min, followed by stopping reaction with 1 M H_2_SO_4_. The absorbance at 450 nm of each well was measured with a microplate reader (Biotek, Shoreline, WA, USA).

For cell binding, CHO-K1/SORT1 cells were incubated with serial diluted antibodies (from 100 nM to 46 pM) at 4 °C for 1 h. Each concentration had three replicates. After washing, cells were incubated with HRP-conjugated goat anti-human IgG at 4 °C for 0.5 h. After washing, TMB was added to cells and the reaction was stopped using 1 M H_2_SO_4_, followed by detecting the absorbance at 450 nm.

### 4.4. Affinity Measurement

The affinity was measured using an OCTET instrument (Sartorius, Göttingen, Germany). The anti-human FC sensors (Sartorius, Göttingen, Germany) were loaded with antibodies (5 µg/mL) for 240 s, followed by association with diluted SORT1 ECD protein for 180 s and dissociations in PBS for 600 s. The equilibrium dissociation constant (KD) was analyzed using Data Analysis 12.0 software (Sartorius, Göttingen, Germany).

### 4.5. Confocal Microscopy

T47D cells were grown on glass coverslip plates at 37 °C for 2 days. Antibodies were added into plates and cells were incubated at 37 °C for 0, 4, or 24 h, followed by incubation at 4 °C for 1 h. After washing, T47D cells were incubated with Fixation and Permeabilization Solution (BD Biosciences, Franklin Lakes, NJ, USA) at 4 °C for 0.5 h, followed by blocking with 2% BSA at room temperature for 0.5 h. The cells were subsequently incubated with Alexa Fluor 647-conjugated goat anti-human IgG (Thermo Fisher, Waltham, MA, USA) to stain human IgG and mouse anti-human CD107a (Proteintech Group, Rosemont, IL, USA) to stain lysosomes at room temperature for 1 h. After washing, cells were incubated with Alexa Fluor 488-conjugated goat anti-mouse IgG (Thermo Fisher, Waltham, MA, USA) to stain mouse IgG at room temperature for 1 h. After washing, DAPI solution (Beyotime, Jiangsu, China) was added to cells to stain nucleus. Confocal images were acquired using Zeiss LSM710 (Zeiss, Oberkochen, Germany). The excitation and emission channels for Alexa Fluor 647 (red) were 633 nm and 683 nm, respectively. The excitation and emission channels for Alexa Fluor 488 (green) were 488 nm and 520 nm, respectively. To detect DAPI (blue), the excitation and emission channels were 405 nm and 452 nm, respectively.

### 4.6. DAR Value Detection of ADC

The DAR value was detected by liquid chromatography–tandem mass spectrometry (LC–MS). After deglycation using PNGase F at 37 °C for 10 min, ADCs were analyzed by LC–MS. The method of liquid chromatography was as follows: Vanquish HPLC (Thermo Fisher, Waltham, MA, USA); MAbPac RP (2.1 × 100 mm, 4.0 μm) (Thermo Fisher, Waltham, MA, USA); solvent A, H_2_O with 0.1% TFA; solvent B, acetonitrile with 0.1% TFA; the gradient was 0–1 min 20% B, 1–11 min 20–45% B, 11–12 min 45% B, 12–18 min 45–20% B, and 18–20 min 20% B; the flow rate was 0.3 mL/min. Mass spectrometry was performed using Thermo Q Exactive HF-X system (Thermo Fisher, Waltham, MA, USA). The DAR value of ADC was calculated using the following equations:

$$\mathrm{DAR}\left(\mathrm{LC}\right)=\frac{\mathrm{Intensity}\mathrm{of}\mathrm{LC}(1\;\mathrm{drug})}{\mathrm{Total}\mathrm{Intensity}\mathrm{of}\mathrm{LC}(0,1\;\mathrm{drugs})}$$

$$\mathrm{DAR}\left(\mathrm{HC}\right)=\frac{\mathrm{Intensity}\mathrm{of}\mathrm{HC}\left(1\;\mathrm{drug}\right)+\mathrm{Intensity}\mathrm{of}\mathrm{HC}\left(2\;\mathrm{drugs}\right)\times 2+\mathrm{Intensity}\mathrm{of}\mathrm{HC}(3\;\mathrm{drugs})\times 3}{\mathrm{Total}\mathrm{Intensity}\mathrm{of}\mathrm{HC}(0,1,2,3\;\mathrm{drugs})}$$

$$\mathrm{DAR}\mathrm{value}=(\mathrm{DAR}(\mathrm{LC})+\mathrm{DAR}\left(\mathrm{HC}\right))\times 2$$

### 4.7. Cytotoxicity Assay

The in vitro cell-killing assay was carried out using MDA-MB-231, MCF-7, MCF-10A, and T47D. Cells were plated in 96-well plates (5000 cells/well). After 1 h incubation, ADCs were added in a serial of concentrations with three replicates per concentration. The concentration of ADCs against MDA-MB-231, MCF-7, and MCF-10A ranged from 300 nM to 137 pM, while the range of concentration was from 100 nM to 46 pM for T47D. This was followed by another 72 h of culture. Cell viability was detected using the Cell Counting Kit 8 (APExBIO, Houston, TX, USA).

To detect the bystander killing activity of ADCs, CHO-K1/SORT1 cells were treated with serial diluted ADCs (from 100 nM to 46 pM). Each concentration had three replicates. After 48 h, cell supernatants were added to Expi293F for incubation another 48 h at 37 °C. Cell viability was then detected using the Cell Counting Kit 8 (CCK8) assay. As a negative control, Expi293F were also directly treated with ADCs at the corresponding concentration.

### 4.8. Flow Cytometry

To detect the surface expression level of targets, tumor cells were incubated with antibodies at 4 °C for 1 h and then stained with Alexa Fluor 647-conjugated goat anti-human IgG (Thermo Fisher, Waltham, MA, USA) for 0.5 h. The mean fluorescence intensity (MFI) of cells was detected by a flow cytometer (Agilent NovoCyte, Santa Clara, CA, USA) and the figure was depicted using FlowJo software10 (BD Biosciences, Franklin Lakes, NJ, USA).

pHrodo^TM^ iFL IgG Labeling Reagents (Thermo Fisher, Waltham, MA, USA) was used to detected the internalization of antibodies. Antibodies (10 nM) were premixed with pHrodo^TM^ iFL green-labeled Fab fragments (40 nM) for 5 min, followed by incubation with T47D for 24 h at 37 °C. The MFI of cells was then measured using flow cytometry.

### 4.9. Xenograft Tumor Growth Model

To evaluate the in vivo efficacy of ADCs, M-NSG mice (females, 6 weeks old) were procured from Shanghai Model Organisms Center, Inc. (Shanghai, China) and BALB/c nude mice (females, 6–8 weeks old) were procured from Shanghai Lab. Animal Research Center (Shanghai, China). A total of 1 × 10^7^ MDA-MB-231 cells were injected via subcutaneous in the right backs of the mice. When the tumor volume reached 100–200 cm^3^, all mice were randomly divided into groups (n = 5 per group). Mice in each group were injected via intravenous with different samples for one dose or two doses on day 0 and day 7. The tumor size and body weight of each mouse from the different groups were measured twice a week until the end of the study. The tumor volume was calculated according to the formula: tumor volume (mm^3^) = longer diameter × (shorter diameter)^2^/2.

### 4.10. Acute Toxicity

The acute toxicity of ADCs in vivo was evaluated using BALB/c mice (females, 6–8 weeks old). Animals were randomly assigned into five groups (n = 5 per group), followed by intravenous injection with one dose of 8D302-MMAE (50 mg/kg), 8D302-MMAE (100 mg/kg), 8D302-DXd (50 mg/kg), 8D302-DXd (100 mg/kg), and PBS (vehicle control), respectively. The body weight of each mouse was measured every day.

### 4.11. Pharmacokinetics Study

The Pharmacokinetics study was performed using BALB/c mice (females, 6–8 weeks old). Two groups (n = 3 per group) of mice was injected via intravenous with one dose of 8D302-MMAE (5 mg/kg) and 8D302-DXd (5 mg/kg), respectively. Serum samples were collected at 0.1, 2, 24, 72, 168, 240, and 336 h post dose. The serum concentrations of total ADCs and total antibody were detected using ELISA. The protocol of ELISA was as follows: 1 µg/mL SORT1 ECD protein were coated on 96-well immunoplates and incubated overnight at 4 °C. After washing, the plate was blocked with 1% BSA, followed by incubation with diluted serum (from 100 to 12,500 fold dilutions) and serial diluted ADCs (from 1000 ng/mL to 105 pg/mL) at 37 °C for 1 h. For the detection of total antibodies, the plates were incubated with HRP-conjugated goat anti-human IgG (Sigma, Taufkirchen, Germany) at 37 °C for 0.5 h after washing. For the detection of total ADCs, the plates were washed and incubated with mouse anti-DXd antibodies (Genscript, Nanjing, China) or mouse anti-MMAE antibodies (Genscript, Nanjing, China) at 37 °C for 1 h, followed by washing and then incubation with HRP-conjugated goat anti-mouse IgG (Sigma, Taufkirchen, Germany) at 37 °C for 0.5 h. After washing, TMB was added. Plates were incubated for 5 min and reactions were stopped by 1 M H_2_SO_4_. The absorbance at 450 nm was measured using a microplate reader (Biotek, Shoreline, WA, USA).

### 4.12. Statistical Analysis

Data analysis was performed using GraphPad Prism 8.0 Software. Group data were reported as mean ± SD or mean ± SEM. *p* values were calculated using *t* test analysis. Group discrepancies were considered statistically significant if *p* < 0.05.

## 5. Conclusions

In summary, our data support the fact that SORT1 can be an attractive target for ADC development because of its upregulated expression in tumor cells and high turnover. Although 8D302-MMAE showed higher anti-tumor activity in vitro and in vivo, the better safety profile of 8D302-DXd may enable it to be a promising therapeutic agent for the treatment of patients with solid cancers, especially breast cancers with SORT1-high expression.

## Data Availability

The data presented in this study are available on request from the corresponding author.

## Supplementary Materials

The following supporting information can be downloaded at: https://www.mdpi.com/article/10.3390/ijms242417631/s1.

## Abbreviations

ADC	Antibody–drug conjugate
PDC	Peptide–drug conjugate
FBS	fetal bovine serum
FDA	U.S. Food and Drug Administration
MTD	maximal toxic dose
CDR	complementary determining region
MMAE	monomethylauristatin E
LC–MS	Liquid chromatography–tandem mass spectrometry
MFI	mean fluorescence intensity
