# Supplementary material for: Generation and Characterization of SORT1-Targeted Antibody–Drug Conjugate for the Treatment of SORT1-Positive Breast Tumor
Source: Int J Mol Sci. 2023 Dec 18;24(24):17631. doi: 10.3390/ijms242417631 (PMC10743877; doi:10.3390/ijms242417631)
Supplement: Supplementary file 1 [file ijms-24-17631-s001.zip › ijms-2735268-supplementary.pdf]

Supplementary Figures

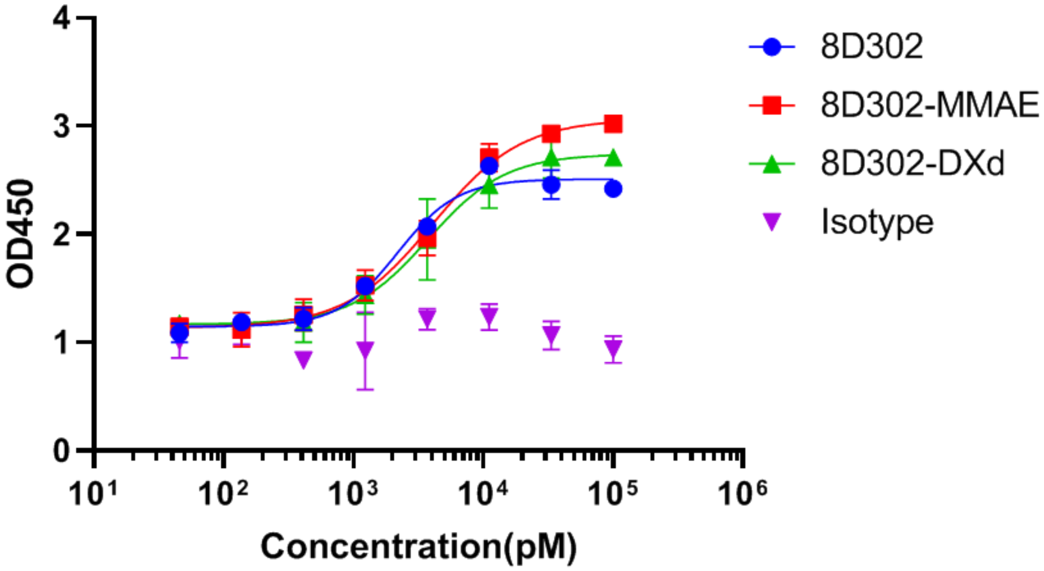

Figure S1. Comparison of cell binding activity between 8D302 and 8D302-ADCs. Data are presented as mean±SD from 3 independent experiments and EC_{50} was calculated using GraphPad Prism software.

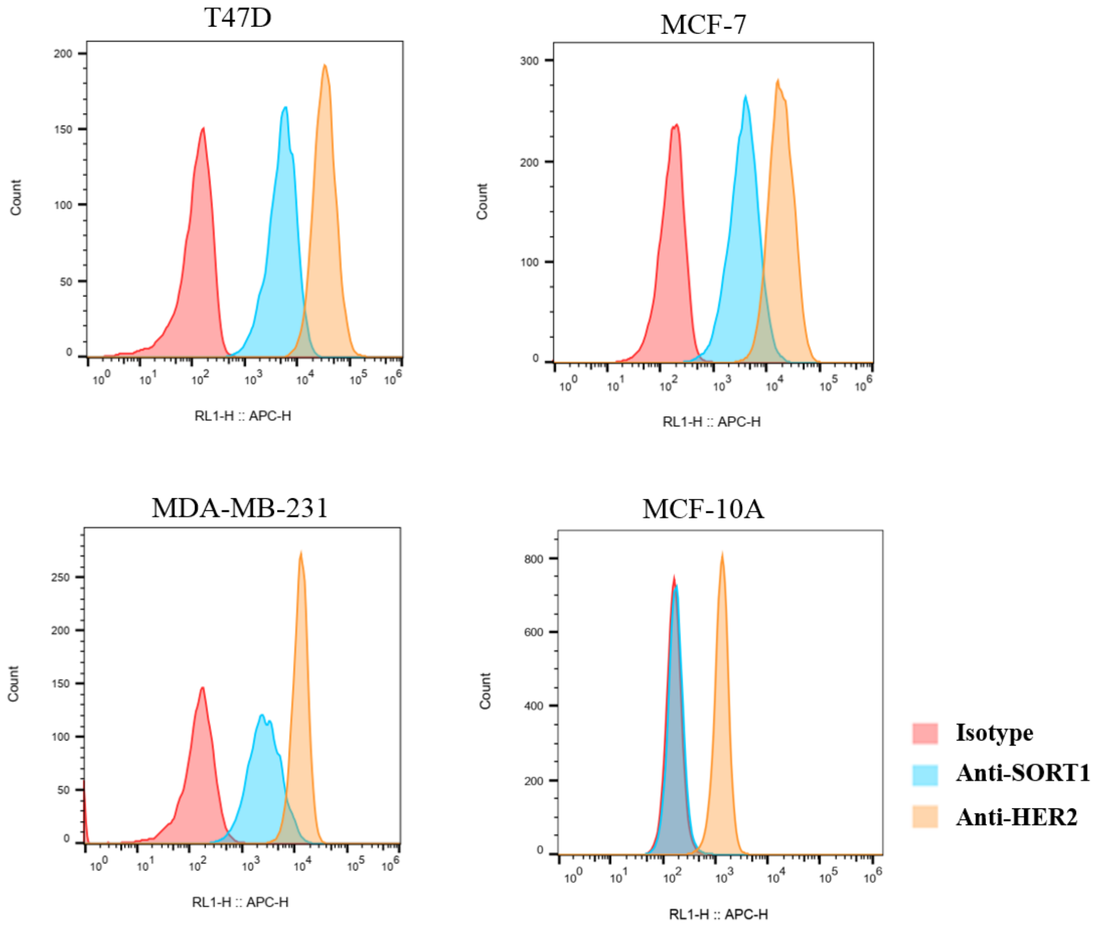

Figure S2. **Evaluation of SORT1 and HER2 expression level on the surface of breast cells.**

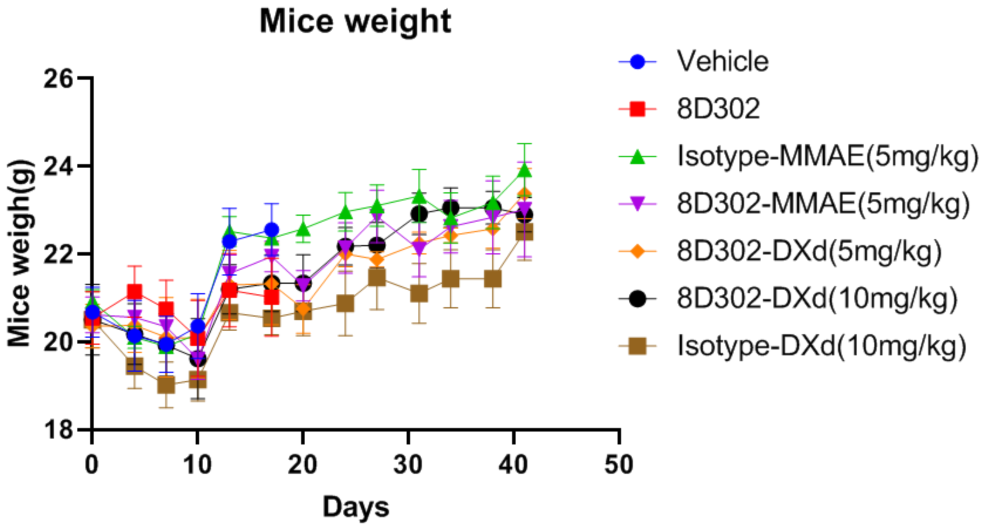

Figure S3. The body weight of tumor-bearing mice. Data are presented as mean±SEM from five mice.
